# Difficult airway associated with bifid glottis and coexistent subglottic stenosis in a patient with Pallister–Hall syndrome: a case report

**DOI:** 10.1186/s40981-018-0158-1

**Published:** 2018-02-23

**Authors:** Yukimura Oe, Kohei Godai, Mina Masuda, Yuichi Kanmura

**Affiliations:** Department of Anesthesiology and Critical Care Medicine, Graduate School of Medical and Dental Sciences, Kagoshima University, 8-35-1 Sakuragaoka, Kagoshima, 890-8520 Japan

**Keywords:** Bifid glottis, Pallister–Hall syndrome, Subglottic stenosis

## Abstract

**Background:**

Pallister–Hall syndrome is a rare disorder characterized by hypothalamic hamartoma, hypopituitarism, bifid epiglottis, and micrognathia.

**Case presentation:**

We describe the airway management under general anesthesia of a 15-year-old female with Pallister–Hall syndrome whose airway was compromised with bifid epiglottis and acquired subglottic stenosis. The three options considered for airway management were tracheal intubation, a supraglottic device, and surgical tracheotomy. Tracheal intubation provides a secured airway, but extubation can be difficult. A supraglottic device minimizes airway injury, but it does not completely protect the airway from aspiration.

**Conclusions:**

The patient’s airway was successfully managed using a supraglottic device with aspiration prophylaxis. Airway management devices should be selected according to each patients’ individual circumstances.

**Electronic supplementary material:**

The online version of this article (10.1186/s40981-018-0158-1) contains supplementary material, which is available to authorized users.

## Background

Pallister–Hall syndrome (PHS) is a rare disorder with a wide spectrum of severity that is characterized clinically by hypothalamic hamartoma, hypopituitarism, bifid epiglottis, imperforate anus, and polydactyly [1, 2]. Airway management in patients with PHS is challenging due to craniofacial anomalies such as micrognathia, hard palate malformation, cleft larynx, gingival cysts, bifid epiglottis and uvula, and mandibular hypoplasia. Patients with PHS are predisposed to aspiration or choking, as the bifid epiglottis provides incomplete airway separation during swallowing.

Subglottic stenosis is stenosis below the glottis and above the first tracheal ring [3]. The incidence of subglottic stenosis has been reported to be less than 0.63% [4]. There are two types of subglottic stenosis: congenital and acquired. Acquired stenosis accounts for the majority (75–95%) of subglottic stenosis cases [3, 4]. The most common cause of subglottic stenosis is prolonged endotracheal intubation [5]. There are numerous reports which describe airway management in patients with subglottic stenosis [6–8]. It is challenging for anesthesiologists to manage airways with subglottic stenosis that also have other airway abnormalities. The use of a supraglottic airway device (SAD) is the preferred choice in managing patents with subglottic stenosis without other airway abnormalities. The airway abnormalities other than subglottic stenosis make it difficult to manage the airways. Anesthesiologists might be reluctant using SAD in patients with the risk of aspiration [9]. Herein, we describe the airway management of a female pediatric patient with PHS, in whom the airway was compromised with bifid epiglottis and acquired subglottic stenosis.

## Case presentation

A 15-year-old female (height, 140 cm; weight, 34 kg) was scheduled for endoscopic treatment of vesicoureteral reflux using dextranomer. The patient had been diagnosed with PHS as a neonate. She had undergone hypothalamic tumor resection as a neonate, repair of imperforate anus at 8 months of age, and gamma knife treatment for hypothalamic tumor recurrence at 11 years of age. Prolonged intubation (2–4 weeks) had occurred after the first and second surgeries due to difficult extubation. Respiratory failure after tracheal extubation occurred several times, without clear reasons. The reason why the difficult extubation had happened was not clear. The anesthetic record showed that tracheal intubation was difficult due to micrognathia and bifid epiglottis. The largest uncuffed endotracheal tube that could be inserted into the trachea when the patient was 11 years old was a 4.0-mm ID. The patient had undergone a gastrostomy due to chronic aspiration. She was also taking medications for hypopituitarism and seizures. She had multiple allergies including egg, latex, and aminophylline. Pre-anesthetic examination of the patient showed that she had micrognathia. Stridor was heard over the trachea. We judged that it would be difficult to obtain the patient’s cooperation with regional anesthesia, as she had mild mental retardation and hearing difficulty. Hence, we selected general anesthesia for the surgery. The patient was planning to receive resection of residual hypothalamic tumor in a few years after the endoscopic treatment of vesicoureteral reflux.

The major anesthetic concern was airway management. Pre-anesthetic problems included (1) difficult intubation due to micrognathia and bifid epiglottis, (2) predisposition to aspiration, and (3) airway stenosis due to prolonged intubation during infancy. We had three options to secure the patient’s airway during the operation: tracheal intubation using video laryngoscopy or fiberoptic bronchoscopy, SAD, or surgical tracheotomy. We elected to use a SAD. Because we wanted to investigate the patient’s airway closely, we decided to observe the vocal cords using a muscle relaxant to minimize the risk of laryngospasm. The neurosurgeons treating the patient were planning to perform resection of the residual brain tumor in a few years. Hence, it seemed important for us to check the patient’s airway before the residual brain tumor resection.

**Fig. 1:**
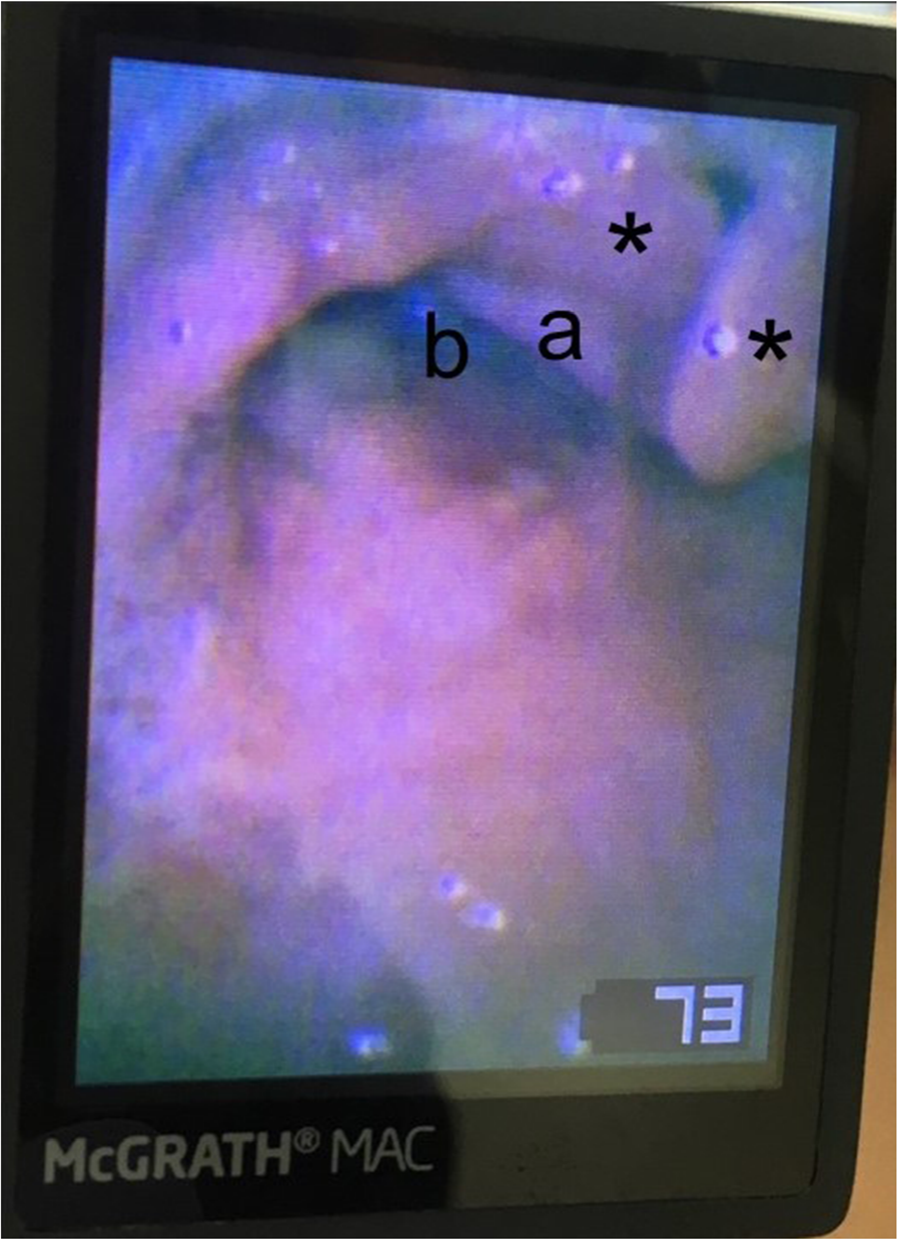
Bifid epiglottis was seen using video laryngoscopy. Bifid epiglottis (*), corniculate cartilage (a), and esophagus (b)

**Fig. 2:**
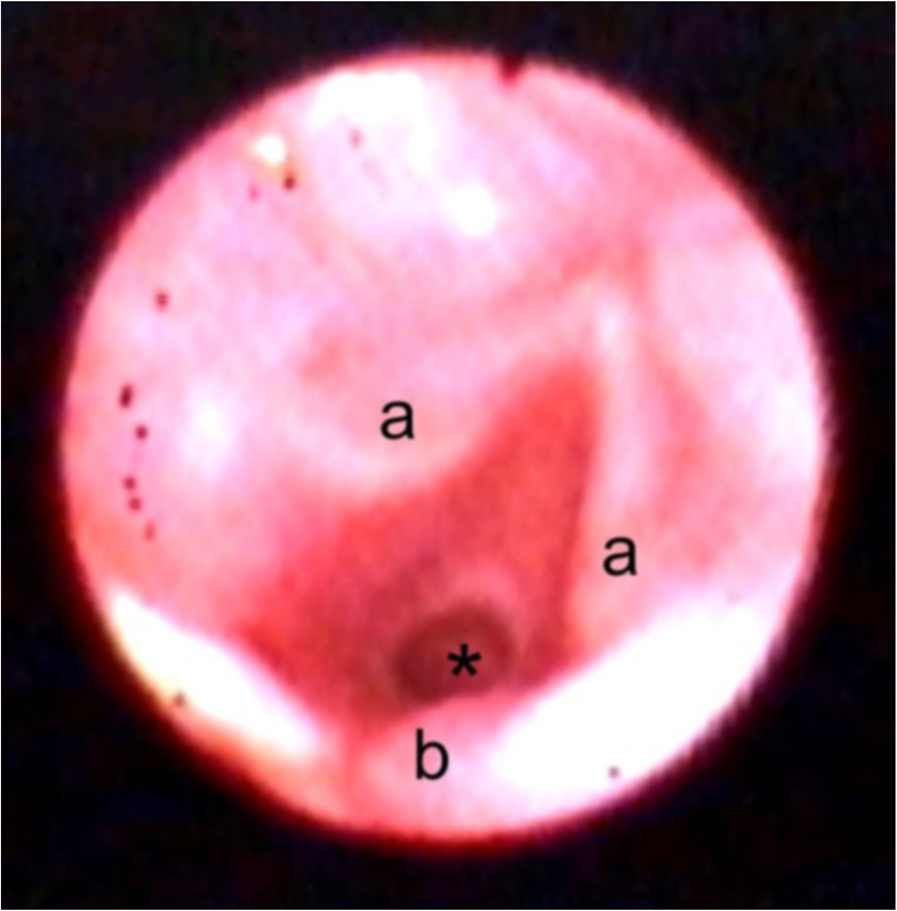
Subglottic stenosis was observed using fiberoptic bronchoscopy. Subglottic stenosis (*), vocal cords (a), corniculate cartilage (b)


Preoperatively, the patient was fasted for 8 h with intravenous hydration. Metoclopramide (10 mg) and an H_2_-blocking agent (ranitidine 50 mg) were administered intravenously to minimize the risk of aspiration. The equipment for difficult airway management was prepared, and two anesthesiologists were present in the operation room. On arrival at the operating room, pulse oximetry, electrocardiography, and noninvasive blood pressure monitoring were established. General anesthesia was induced with intravenous propofol (50 mg) and fentanyl (25 μg). Mask ventilation was successful. Rocuronium (20 mg) was administered to facilitate positive pressure ventilation. Because we needed to investigate the patient’s airway for future surgery, we observed the epiglottis and vocal cords using a McGRATH MAC video laryngoscope with an X-blade (Medtronic, Minneapolis, MN) and confirmed the bifid epiglottis (Fig. 1). A laryngeal mask airway (size 2.5) was inserted, and the cuff was inflated with air (10 ml). We evaluated the vocal cords and trachea using fiberoptic bronchoscopy and visualized the subglottic stenosis (Fig. 2, video). The subglottic stenosis was the reason that only a small-sized endotracheal tube could be inserted when the patient was 11 years old. Anesthesia was maintained by sevoflurane 2.5% and remifentanil (0.2–0.5 μg/kg/min) and intermittent doses of rocuronium. The operation time was 27 min. For postoperative analgesia, acetaminophen (500 mg) was administered intravenously. After confirming spontaneous recovery of a train-of-four ratio to 100%, we decided not to antagonize the rocuronium, as the patient had multiple allergies and was considered high risk for sugammadex allergy. The laryngeal mask airway was removed after recovery of spontaneous breathing. The postoperative course was uneventful, and the patient was discharged the day after the procedure.

Additional file 1: Video. Subglottic stenosis was observed using fiberoptic bronchoscopy. (MP4 88,406 kb)


## Discussion

This case report describes the challenging airway management in a patient with PHS. Tracheal intubation in PHS patients is difficult because of the clinical features associated with the disease, including micrognathia, bifid epiglottis, and mandibular hypoplasia [2]. The bifid epiglottis predisposes to aspiration. Our patient had acquired subglottic stenosis, which made it more difficult to manage her airway during anesthesia. Both tracheal intubation and SAD usage have advantages and disadvantages. Airway management devices must be selected in advance according to the characteristics of the patient and the surgery. In the present case, we successfully managed the patient using a SAD.

We had three options to secure the patient’s airway during the operation: tracheal intubation using video laryngoscopy or fiberoptic bronchoscopy, SAD, or surgical tracheotomy. Each option had advantages and disadvantages. Tracheal intubation provides a secured airway against aspiration; however, this method may lead to prolonged intubation and/or subsequent surgical tracheotomy because of extubation difficulty. It seemed that intubation using video laryngoscopy or fiberoptic bronchoscopy was not difficult. It also seemed that the extubation after tracheal intubation would be difficult, as tracheal intubation might lead to mucosal edema. It is well known that intubation through the stenotic lesion may cause mucosal edema and further narrowing the airway [10]. The use of a SAD can minimize tracheal injury during anesthesia, although the use of a SAD does not provide complete aspiration protection. Surgical tracheotomy is the most invasive option, although it reduces the patient’s respiratory workload compared with tracheal intubation or the use of a SAD. Surgical tracheotomy should not be considered first choice due to the invasive feature. Regional anesthesia is ideal for anesthetic management of patients with subglottic stenosis if general anesthesia is not necessary [10]. Regional anesthesia was not possible in the present case, as the patient had difficulty in communication because of mental retardation and a hearing problem. When general anesthesia is necessary, we need to evaluate the possibility of mask ventilation under general anesthesia. If mask ventilation seems impossible, extracorporeal membrane oxygenation should be considered. In the present case, the previous anesthetic record showed that mask ventilation of the patient was possible. Therefore, we decided to induce general anesthesia.

As the bifid epiglottis does not provide complete protection against aspiration, PHS patients may have lung damage due to repeated aspiration pneumonia [11]. Careful pre-anesthetic airway evaluation is therefore crucial in PHS patients. Anesthetic considerations for management of patients with PHS include endocrine and neurological derangements [2]. PHS patients may be taking corticosteroids and other hormonal replacement therapy due to hypopituitarism; stress doses of corticosteroids need to be administered perioperatively [12]. As PHS is associated with neurological conditions such as hypothalamic hamartoma and seizures, anticonvulsant therapy should be continued perioperatively [13]. Intracranial pressure may be elevated in patients with hypothalamic hamartoma, and so hypoventilation should be avoided to minimize the risk of further increasing the intracranial pressure [14]. There may be difficulty with cooperation in PHS patients with mental retardation. We avoided sugammadex in the present case after confirming spontaneous recovery of the neuromuscular blockade, as anaphylaxis is a rare but serious adverse reaction to sugammadex [15].

## Conclusions

We report the challenging airway management associated with bifid glottis and coexistent subglottic stenosis in a patient with PHS. The possibility of subglottic stenosis should be evaluated preoperatively. Airway management devices should be selected according to each patients’ individual circumstances.

## Abbreviations

PHS: Schwartz–Jampel syndrome
SAD: Supraglottic airway device
